# Experimental comparison between ZnO and MoS_2_ nanoparticles as additives on performance of diesel oil-based nano lubricant

**DOI:** 10.1038/s41598-020-62830-1

**Published:** 2020-04-02

**Authors:** Seyed Borhan Mousavi, Saeed Zeinali Heris, Patrice Estellé

**Affiliations:** 1 Faculty of Chemical and Petroleum Engineering, University of Tabriz, Tabriz, Iran; 2 Univ Rennes, LGCGM, EA3913, F-35000 Rennes, France

**Keywords:** Chemical engineering, Nanoparticles, Nanoparticles

## Abstract

This study compares the tribological and thermophysical features of the lubricating oil using MoS_2_ and ZnO nano-additives. The average size of MoS_2_ and ZnO nanoparticles were 90 nm and 30 nm, respectively. The nanoparticles were suspended using Triton X-100 in three different concentrations (0.1, 0.4 and 0.7 wt.%) in a commercial diesel oil. Tribological properties such as mass loss of the pins, friction coefficient, and worn surface morphologies and thermophysical properties such as viscosity, viscosity index, flash point and pour point of resulting nano lubricant were evaluated and compared with those of pure diesel oil. The tribological behavior of nano lubricants was evaluated using a pin-on-disc tribometer. The worn surface morphologies were observed by scanning electron microscopy. The overall results of this experiment reveal that the addition of nano-MoS_2_ reduces the mass loss values of the pins in 93% due to the nano-MoS_2_ lubricant effect. With 0.7 wt.% in nanoparticles content, the viscosity of MoS_2_ and ZnO nano lubricants at 100 °C increased by about 9.58% and 10.14%, respectively. Pure oil containing 0.7 wt.% of each nanoparticle increased the flash point because of its small size and surface modifying behavior compared to the pure oil. Moreover, the addition of ZnO nanoparticles with pure oil lubricant is more suitable than MoS_2_ nanoparticles for improving the thermophysical properties of pure oil.

## Introduction

Friction is one of the important factors of energy loss in mechanical parts. Lubrication plays a very important role in the correct, continuous and economic movement. The lack of proper lubrication of machinery reduces the mechanical efficiency; additionally, it will result in excessive erosion and exhaustion. The choice of suitable lubricant has a special effect on the performance of the machinery. The major losses that occur in a car engine can be friction between moving parts. The main role of the lubricant is to keep the surface of the two metals moist, that separates them from each other by creating a suitable layer on the surfaces with friction, and the heat and created abrasive particles will be eliminated. Friction is minimized and prevents wear^1–3^. A good lubricant should have a high viscosity index (VI), high flash point and low pour point. Nano-additive oil-based lubricants exhibit excellent lubrication performance for tribological applications^4,5^. The most important advantage of using nano-additives in lubricants is their small size, which acts as a roller on the interface, leads to an increase in contact surfaces and reduces the wear and friction^6^. Depending on their structural properties, their size and concentration in the base fluid, nano-additives can improve the tribological and thermophysical properties of the base fluid^7–9^. The major problem with the use of nano-additives is agglomeration in lubrication mechanisms^10^. Recently, the use of MoS_2_ nanoparticles as solid lubricants has been highly regarded due to their proper lubrication properties. The crystalline structure of the MoS_2_ nanoparticles is hexagonal. Its intrinsic lubricity properties relate to the vast space and weak Van der Waals forces between the S-Mo-S sandwich layers and the pure positive charge on the surface, which results in the spread of electrostatic repulsion. Layers are located with the weak molecular forces and can easily slip on each other^11–15^. ZnO nanoparticles are highly regarded for its high surface energy, good dispersion properties, and unique electrical and thermal conductivity. Adding ZnO nanoparticles to base oils can improve its anti-wear feature by creating a lubricant film and reducing the friction of moving surfaces. One of the defects of these nanoparticles is their poor solubility and high surface energy, that can result in the agglomeration of particles^16–18^.

Rajendhran *et al*.^6^ studied the effect of MoS_2_ nano-additives on the frictional features of the lubricating oil. The results revealed that pure oil containing 0.5 wt.% nano-additives could strikingly improve the tribological properties. Ali *et al*.^8^ suspended diverse nanoparticles using oleic acid in the base oil at different concentration in order to investigate the tribological properties of the prepared nano lubricants. They concluded that the kinematic viscosity and the friction coefficient of the nano lubricants declined as a result of the incorporation of the nanoparticles. Katiyar *et al*.^19^ developed magnetic paraffin-based nanofluid with different concentrations of Fe-Ni nanoparticles and studied the effect of a magnetic field and nanoparticle concentrations on rheological properties. They found that the viscosity and shear stress of a magnetic nanofluid with more than 8 wt.% Fe-Ni nanoparticles increased significantly with concentration. Singh *et al*.^20^ found that the thermal conductivity of base fluid is improved with the addition of diverse nanoparticles. Ali *et al*.^21^ reported that the thermophysical behavior and lubrication features of the pure engine oil is enhanced with the addition of Al_2_O_3_ and TiO_2_ nanoparticles. Rasheed *et al*.^22^ concluded that using graphene nanoparticles as lubricant additives enhanced the lubricity function and thermal conductivity of the engine oil. Numerous studies have been conducted to investigate the tribological features and thermophysical behaviors of MoS_2_ nano-additive oil-based lubricants. The results proved that the lubricity characteristics of the prepared nanofluids is strikingly improved with the corporation of MoS_2_ nanoparticles^10,23–35^. Cabaleiro *et al*.^36^ studied the thermophysical and rheological characteristics of the ZnO nanofluids. Their findings indicated that thermal conductivity and density of the prepared nanofluids increased with the addition of ZnO nanoparticles. Pastoriza-Gallego *et al*.^37^ investigated the thermophysical properties of ZnO nanoparticles dispersed in ethylene glycol. They concluded that the prepared ZnO nanofluid had higher thermal conductivity and viscosity compared to the pure ethylene glycol. Sanukrishna *et al*.^38^ investigated the thermophysical properties of SiO_2_ nano-additive oil-based lubricants. They showed that the pure lubricant containing nano-SiO_2_ had higher thermal conductivity and viscosity compared to the pure lubricant. Guimarey *et al*.^39^ studied the thermophysical characteristics of the ZrO_2_ nano lubricants. Their findings indicated that the viscosity and density of the prepared nanofluids increased with the addition of ZrO_2_ nanoparticles. Ingole *et al*.^40^ evaluated the friction reduction and anti-wear properties of the TiO_2_ nanofluids. They proved that the addition of TiO_2_ nanoparticles to the base oil improved tribological performances. Yang *et al*.^41^ investigated the titanium alloys tribological feature lubricated by various fluids. They claimed that self-emulsifying ester (SEE) showed better friction decrease efficiency on titanium alloys than the frequently applied paraffin. Investigations of the lubrication behavior and thermophysical properties of silica nanoparticles proved that the addition of silica nanoparticles to the base fluid can improve the anti-friction and anti-wear features^42–44^. Chen *et al*.^45^ found that the addition of CuS nanorods as additives to lubricating oil causes a substantial improvement in the tribological properties in comparison with the pure oil. Jukić *et al*.^46^ studied the rheological features of polyolefin copolymer (OCP) and terpolymer poly (styrene-co-dodecyl methacrylate-co-octadecyl methacrylate) (PSAMA) in lubricating oil. The results revealed that the viscosity index of the solution increased with the addition of PSAMA and also can decrease the pour point. The tribological properties of new MoS_2_ nanoparticles prepared by seed-assisted solution technique were assessed by Njiwa *et al*.^47^ and a significant improvement was shown in the tribological properties compared with pure oil. They also reported that the lubrication contact is linked to critical size of the particles. Aladag *et al*.^48^ studied the rheological behaviors of Al_2_O_3_ and CNT nanofluids. The results showed that the prepared Al_2_O_3_ and CNT nanofluids are non-Newtonian and Newtonian, respectively. They further reported that a viscosity hysteresis phenomenon is seen for both nanofluids. Ali *et al*.^49^ investigated Al_2_O_3_/TiO_2_ nanoparticles effects on tribological properties of engine oil. They asserted that adding Al_2_O_3_/TiO_2_ nanoparticles to the base oil can improve fuel consumption and lubricity characteristics. Luo *et al*.^50^ investigated the tribological efficiency of WS_2_ submicron spheres as additives in lubricating liquid. They concluded that WS_2_ submicron spheres improved tribological properties of paraffin liquid. Kumar *et al*.^51^ conducted a study about the tribological characteristics of an oil-in-water emulsion. They dispersed oil droplets in water using different concentrations of anionic surfactant. They concluded that smaller droplets had better tribological properties than bigger ones. They also reported that the optimal range of concentration of surfactant was 0.5–1 mM. Wu *et al*.^52^ reported that the addition of polymeric nanoparticles can decrease the frictional coefficient. Fan *et al*.^53^ prepared highly oxygen/fluorine dual functionalized graphene (OFG) and used them as additives to evaluate the tribological performance of water-based lubricant, proving that they can effectively improve the tribological properties.

This study experimentally compares the effects of MoS_2_ and ZnO nanoparticles on thermophysical and tribological properties of a commercial diesel oil. Furthermore, the impact of the nanoparticle’s concentration on the thermophysical and tribological behavior of nano lubricants is explored. The tribological properties are examined by a pin-on-disc tribometer. Kinematic viscosity, viscosity index, pour point and flash point value of nano lubricants were experimentally evaluated. To determine the friction factor, a system has been designed and constructed. The structure of the nanoparticles and worn surfaces were characterized by scanning electron microscopy (SEM).

## Experimental Details

### Nanoparticles and lubricant

This study aims to improve the lubricity characteristic of pure diesel oil using ZnO and MoS_2_ nano-additives. The SAE-40 monograde diesel oil (Special Super Kian) used as the base oil in the experiments is supplied by Pars oil co, Iran. The ZnO and MoS_2_ nanoparticles were purchased from US Research Nanomaterials and Sigma-Aldrich, respectively. The main characteristics of the nanoparticles and pure diesel oil are presented in Table 1. In order to prepare the nano lubricants by a two-step method, ZnO and MoS_2_ nanoparticles are suspended in pure diesel oil on a diverse weight percentage basis (0.1, 0.4 and 0.7). Triton X-100 (Samchun, China) was used as a surface modifier to provide scattering durability of the additives in pure diesel oil. Numerous studies have revealed that nonionic surfactants, such as the polyoxyethylene octyl phenols (e.g. the Triton X series), do not have any negative effects on the oil quality^54–56^. The amount of Triton X-100 was 50 wt.% of each nanoparticle. In order to attain a steady suspension of nanoparticles in the diesel engine oil, a mechanical stirrer was used at 900 r/min for 200 min. The required amount of each nanoparticle and Triton X-100 was exactly weighed using a precision digital scale (Kern, Germany) and then added to the pure diesel oil. The prepared nano lubricants were then agitated using an ultrasonic shaker (Parsonic 2600 s, Iran) for 45 min to ensure sustainable nano lubricants. The scanning electron microscope (Zeiss, Germany) was used to survey the morphology of the nanoparticles. The X-ray diffractometer (PHILIPS-PW1730, Netherlands) was employed to examine the crystal structures of the ZnO and MoS_2_ nanoparticles. XRD patterns were recorded using Cu- Kα radiation (λ = 1.5406 Å) at 40 kV and 30 mA from 7° to 80° (2θ) at a rate of 3° min^−1^. The crystallite size of ZnO and MoS_2_ were calculated based on each XRD pattern, using Debye–Scherrer’s equation^57^:

1 $${\rm{D}}=\frac{0.9\ast {\rm{\lambda }}}{\beta (\cos \,\theta )}$$

where D, *λ*, *β* and *θ* are the crystallites size (nm), the radiation wavelength, the full width at half of the maximum of the peak (radians) and the angular position of the peak. The transmission electron microscope (TEM, PHILIPS-CM120, Netherlands) was used in order to identify the morphology and size distribution of the ZnO nanoparticles.

**Table 1:** Material characteristics.

Materials	Properties			
Nanoparticles	Purity (%)	Size (nm)	Density (g/cm^3^)	Color
ZnO	99.9	30	5.6	White
MoS_2_	99	90	5.06	Gray/Black
Base Oil	Physical properties			Value
Monograde diesel oil (SAE-40)	Kinematic viscosity, cSt at 40 °C			162.53
	Kinematic viscosity, cSt at 100 °C			14.62
	Density, Kg/cm^3^ at 15 °C			0.893
	Viscosity Index			86.85
	Pour point, °C			−17.8
	Flash point, °C			238

### Viscosity and viscosity index measurement

The kinematic viscosity of prepared samples was measured by Cannon-Fenske Opaque (200) viscometer according to ASTM D-445 standard at atmospheric pressure and at temperatures of 40 °C, 60 °C, 80 °C and 100 °C. In order to investigate the effect of nanoparticles concentration on kinematic viscosity, experiments were carried out at different concentrations (0.1, 0.4 and 0.7 wt.%) and the results were compared with the pure lubricant. For reliability, repeatability and error reduction, each test is repeated three times and the mean value is considered.

In order to calculate the viscosity index of the samples, the following equation was utilized according to the ASTM D-2270:

2 $${\rm{VI}}=\left[\frac{{\rm{L}}-{\rm{U}}}{{\rm{L}}-{\rm{H}}}\right]\,\ast \,100$$

where U, L, and H are the oil’s kinematic viscosity at 40 °C, the values of kinematic viscosity at 40 °C for oils of viscosity index 0 and 100, respectively, which have the same kinematic viscosity at 100 °C as the oil whose viscosity index are tried to be ascertained.

If the basic kinematic viscosity at 100 °C is in the range of 2–70 cSt, L and H values are obtained from the tables of ASTM D-2270. Otherwise, if the kinematic viscosity is greater than 70 cSt, the L and H values are computed as follows:

3 $${\rm{L}}=0.8353\,{{\rm{Y}}}^{2}+14.67\,{\rm{Y}}-216$$

4 $${\rm{H}}=0.1684\,{{\rm{Y}}}^{2}+11.85\,{\rm{Y}}-97$$

where Y is the kinematic viscosity at 100 °C of the oil whose viscosity index is to be determined. The corresponding values of L and H were obtained by linear interpolation if the measured values are not listed in the tables, but are within the range of 2–70 cSt.

The viscosity index was calculated by Eq. (5).

5 $${\rm{VI}}=[(({\rm{antilog}}\,{\rm{N}})-1/0.00715]+100$$

6 $${\rm{N}}=\frac{\log \,{\rm{H}}-\,\log \,{\rm{U}}}{\log \,{\rm{Y}}}$$

### Flash point and pour point measurement

Flash point and pour point of the prepared nano lubricants were experimentally measured based on ASTM D-92 (Cleveland open cup method) and ASTM D-97 respectively, using proper apparatus.

### Tribological tests

**Figure 1:**
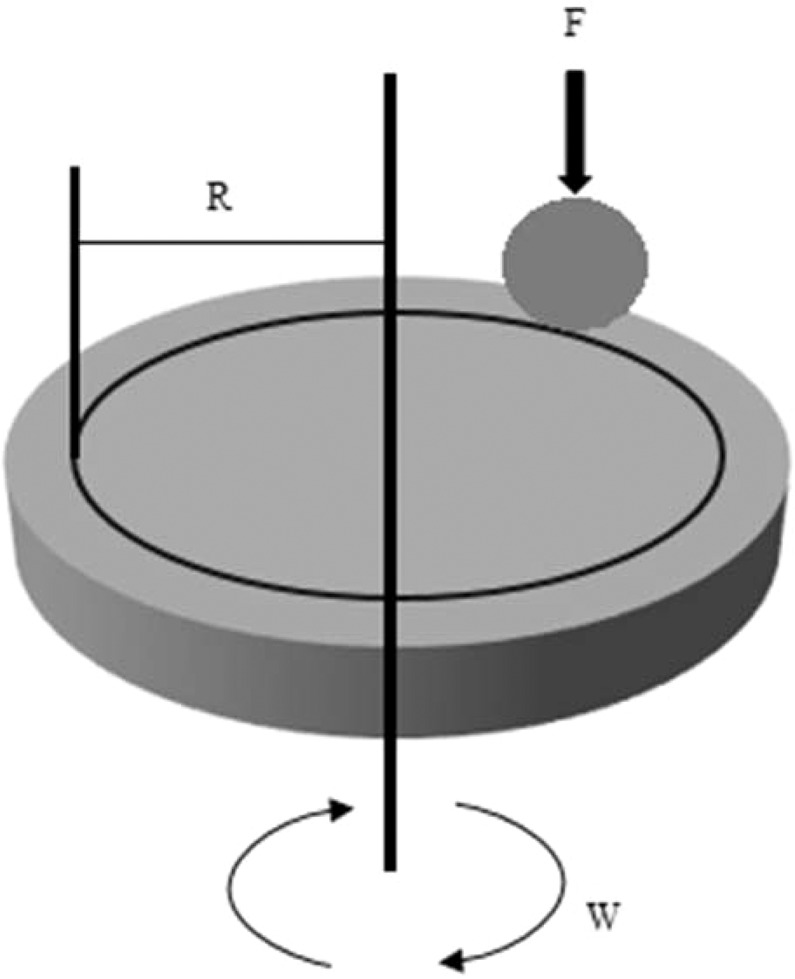
Schematic image of the pin-on-disc apparatus.

The pin-on-disc tribometer (Tajhiz Sanat Nasr, Iran) was used to determine the anti-wear and anti-friction behavior in the presence of ZnO and MoS_2_ nano lubricants and comparison with pure diesel oil. To determine the effect of nanoparticles concentration on anti-wear and anti-friction properties, the experiments were carried out at three concentrations of each nano lubricant. The test was carried out at a load of 75 N, at 150 rpm, at a distance of 1000 m at a temperature of 25 °C and friction coefficient values have been recorded. The mass loss values of the pins are also measured with a precision of 0.0001 gr. Each test is repeated three times, and the average mass loss of pins is calculated. The material of the pins and discs were 52100 steel and MO40 steel, respectively. Figure 1 shows the pin position and wear pattern, wear direction and direction of rotation of the disc. Where F is the normal force on the pin and W is the disk rotational speed.

The worn surfaces morphology is characterized by SEM. Before the worn surfaces analysis, each of the samples was well cleaned with acetone in order to remove the residual lubricant.

### Friction factor measurement

**Figure 2:**
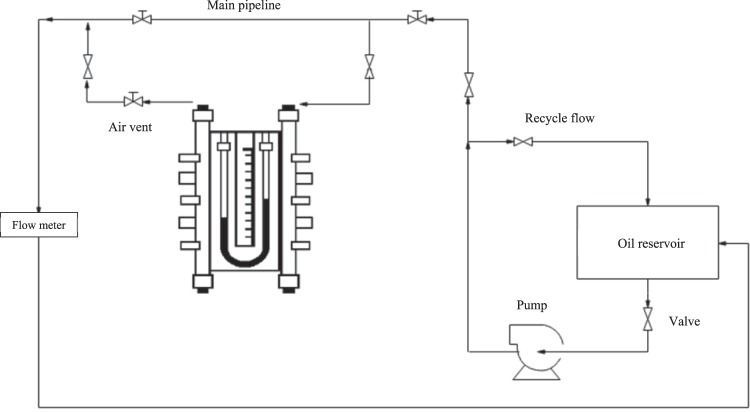
Schematic of the experimental setup.

In order to determine the friction factor and drop of fluid pressure inside the tube in different flow rates, a laboratory system was designed and constructed. The schematic design of the laboratory system is presented in Fig. 2. The laboratory system consists of a circuit that is composed of various sections for measuring the pressure and flow rates. The components of the laboratory system include a fluid reservoir and a diesel pump for the transfer of fluid to the main lines. To adjust the required flow rate, a recycle flow to the reservoir is used. The test section was constructed by using a 1.1 m long smooth copper pipe with an inner diameter of 9 mm, and along the pipe, the air vent valves were used to evacuate the bubbles in the oil to minimize the error of the test.

## Data analysis

The theoretical friction factor (f) for laminar flow and Reynolds number are obtained as^58,59^:

7 $$f=\frac{64}{Re}$$

8 $$Re=\frac{uD}{\nu }$$

where *v*, *u* and *D* are kinematic viscosity, velocity and diameter of the pipe as a characteristic length and Re is Reynolds number.

Velocity and flow rate determined by Eqs. (9) and (10), respectively^60^.

9 $$u=\frac{Q}{A}$$

10 $$Q=\frac{V}{t}$$

where *Q*, *A*, *V*, and *t* are flow rate, cross-section area of the pipe, volume and time.

The experimental pressure difference is obtained from the U-shaped manometer and the height change of oil and carbon tetrachloride. Theoretical (*∆*p_th_) and experimental (*∆*p_exp_) pressure differences were determined by Eqs. (11) and (12), respectively^61^.

11 $${{\Delta }{\rm{p}}}_{{\rm{th}}}={\rm{f}}\frac{{\rm{L}}}{{\rm{D}}}\ast \frac{{{\rm{\rho }}{\rm{u}}}^{2}}{2}$$

12 $${{\Delta }{\rm{p}}}_{\exp }={{\rm{h}}}_{1}{\rm{g}}{{\boldsymbol{\rho }}}_{{\rm{ccl}}4}\mbox{--}{{\rm{h}}}_{2}{\rm{g}}{{\boldsymbol{\rho }}}_{{\rm{oil}}}$$

where L, ***ρ***, g, h and ccl4 are the pipe length, density, gravity, height of liquid in manometer and carbon tetrachloride respectively.

Since the measured parameters have different accuracy, the effect of the measurement error of these parameters on the results is investigated here.

As stated above, the Reynolds number is defined as ${\rm{Re}}=\frac{{\rm{uD}}}{{\rm{\nu }}}$, and the resulted error from the Reynolds number is expressed as follows^62^:

13 $${E}_{Re}=\sqrt{{({E}_{u})}^{2}+{({E}_{D})}^{2}+{(-{E}_{{\rm{\nu }}})}^{2}}$$

Given that ${\rm{u}}=\frac{{\rm{Q}}}{{\rm{A}}}$, *E*_*U*_ is calculated as follows:

14 $${E}_{U}=\sqrt{{({E}_{Q})}^{2}+{(-{E}_{A})}^{2}}$$

Also, the caused error by the volumetric flow rate defined as ${\rm{Q}}=\frac{{\rm{V}}}{{\rm{t}}}$ that is determined by the Eq. (15).

15 $${E}_{Q}=\sqrt{{({E}_{V})}^{2}+{(-{E}_{t})}^{2}}$$

The error of the friction coefficient measurement is equal to the error associated with the Reynolds number.

16 $${E}_{f}={E}_{Re}$$

Therefore, the maximum friction factor error in the experiments is obtained at 1.1%.

## Results and discussion

### Characterization of MoS_2_ and ZnO nanoparticles

**Figure 3:**
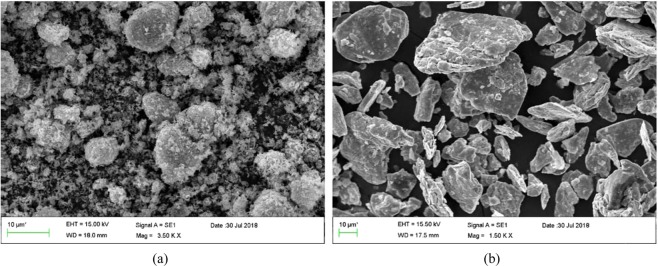
SEM images of (**a**) ZnO nanoparticles, (**b**) MoS_2_ nanoparticles.

**Figure 4:**
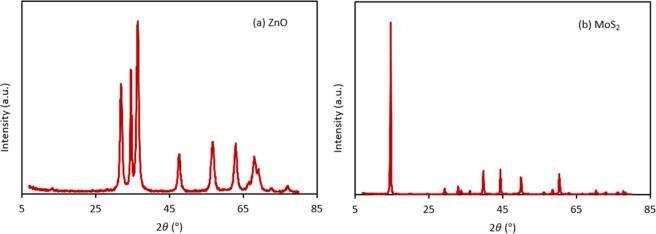
XRD patterns of (**a**) ZnO nanoparticles, (**b**) MoS_2_ nanoparticles.

**Figure 5:**
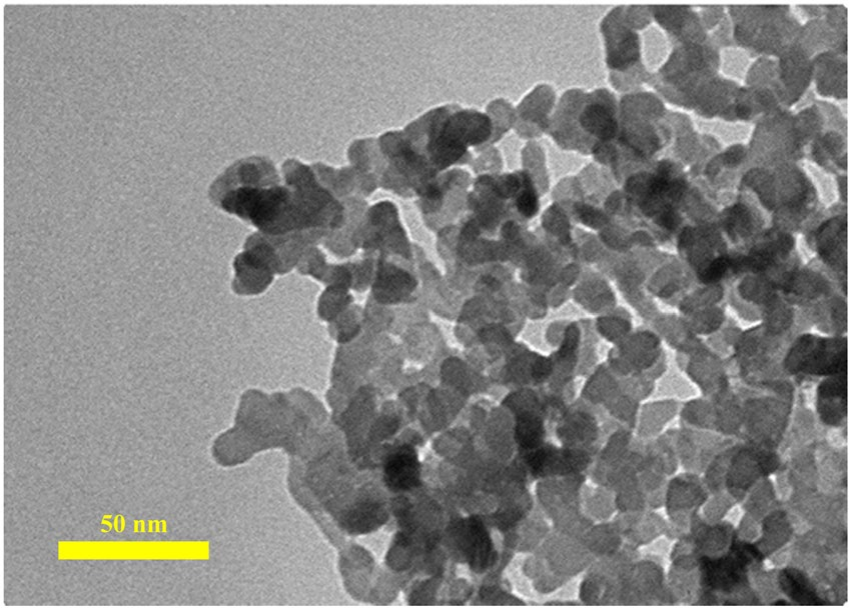
TEM image of ZnO nanoparticles.

**Figure 6:**
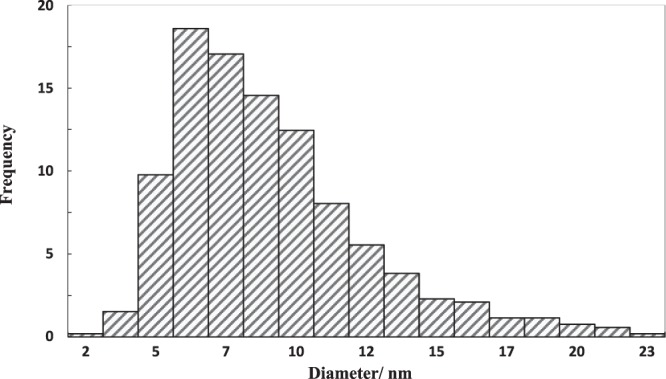
Size distribution of ZnO nanoparticles.

Figure 3 shows the SEM images of the nanoparticles. The SEM micrograph reveals that ZnO nanoparticles have an almost spherical shape with an average diameter of 30 nm. (Fig. 3a). According to the SEM image (Fig. 3b), the MoS_2_ nanoparticles have a platelet-like shape with an average diameter of 90 nm. The XRD pattern of the ZnO nanoparticles (Fig. 4a) exhibited diffraction peaks at 2*θ* = 31.88, 34.58, 36.43, 47.73, 56.73, 62.93, 66.83, 68.03 and 69.13 corresponding to the (100), (002), (101), (102), (110), (103), (200), (112) and (201) planes of ZnO, consistent with the corresponding standard card (JCPDS card number 36–1451)^63^. The MoS_2_ (Fig. 4b) showed diffraction peaks at 2*θ* = 14.63, 29.18, 32.88, 39.73, 44.38, 50.13, 60.48 and 70.23 corresponding to the (002), (004), (100), (103), (104), (105), (112) and (200) crystal planes of the MoS_2_ structure according to the reference data of JCPDS card number 37–1492^64^. No impurity peaks or other phases were observed. The crystallite size of ZnO and MoS_2_ determined from the main peak breadth in the XRD pattern by Eq. (1) were 14.2 nm and 32.6 nm, respectively. The TEM image of the ZnO nanoparticles (Fig. 5) revealed that the ZnO nanoparticles tended to agglomerate and did not permit identification of a single particle owing to the high surface energy of nanoparticles. Furthermore, TEM indicates that the ZnO nanoparticles are approximately spherical and the average diameter of the nanoparticles is 10.3 nm (Fig. 6), in good agreement with the value obtained from the Scherrer formula.

The nano lubricant properties depend on base fluid (here diesel oil), nature of nanoparticles (here MoS_2_ and ZnO), morphology, shape, size and concentration. In this study, we focused only on the concentration of nanoparticles on diesel oil properties, considering two types of nanoparticles. Literature review shows that the nanoparticles smaller than 50 nm in diameter are capable to improve the tribological and thermophysical properties of water based nanofluid, and the reduction in nanoparticle size enhances these properties. However, for nanoparticles smaller than 10 nm in diameter, the opposite effects may be obtained. In this study, the results are reported as a comparison of purchased commercial nanoparticles. In future studies, the effect of nanoparticle size on thermophysical and tribological properties can be investigated as shown^32,65,66^.

### Dispersion stability of the MoS_2_ and ZnO nanoparticles

**Figure 7:**
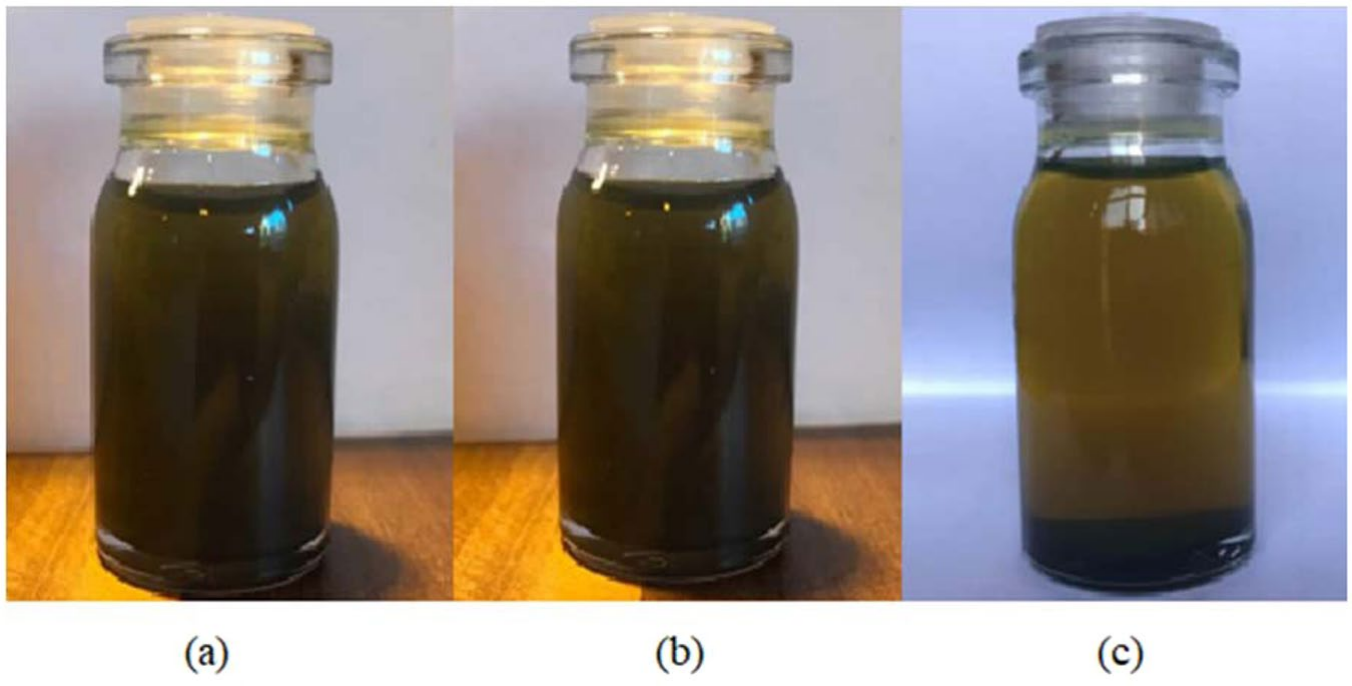
Camera pictures of MoS_2_ nano lubricants (0.1 wt.%) after (**a**) 12 hours, (**b**) 1 day, (**c**) 2 days.

**Figure 8:**
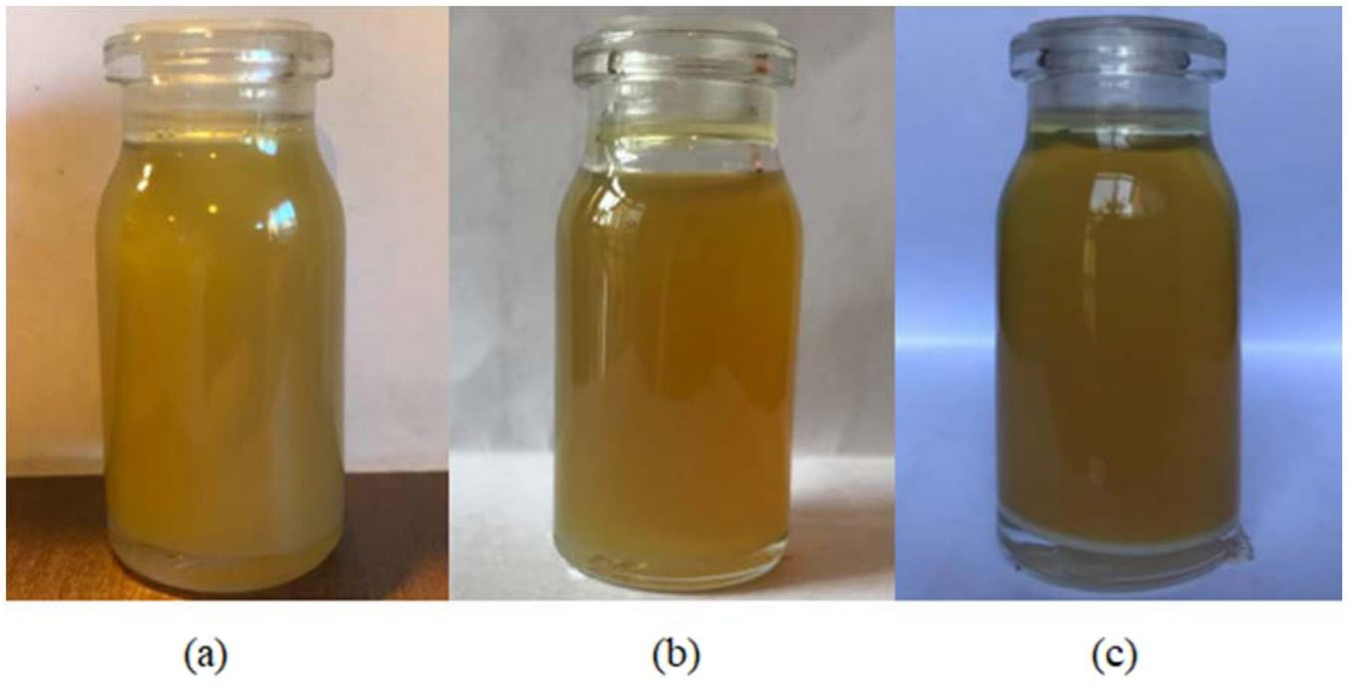
Camera pictures of ZnO nano lubricants (0.1 wt.%) after (**a**) 1 day, (**b**) 5 days, (**c**) 6 days.

The 0.1, 0.4 and 0.7 wt.% nano-additives were mixed with pure diesel oil. For the uniform distribution of nanoparticles and better stability, the prepared nano lubricant was placed in an ultrasonic bath at 25 °C for 45 minutes. Figure 7 shows the stability of MoS_2_ nano lubricants at 0.1 wt.% that is taken at room temperature after 12 hours, 1 day and 2 days. Considering the figure, it is observed that the MoS_2_ nano lubricant has good dispersion stability up to 1 day and no precipitation was observed. After 1 day, precipitation was started and gradually the nano lubricant was cleared. Figure 8 shows the stability of ZnO nano lubricants at 0.1 wt.% that is taken after 1 day, 5 days and 6 days. The prepared ZnO nano lubricant in low concentrations was stable for at least 5 days and at high concentrations was stable for at least 3 days so that no deposition occurs in this range. By comparing Figs. 7 and 8 it can be seen that the stability of ZnO nanoparticles was better than MoS_2_ nanoparticles.

**Figure 9:**
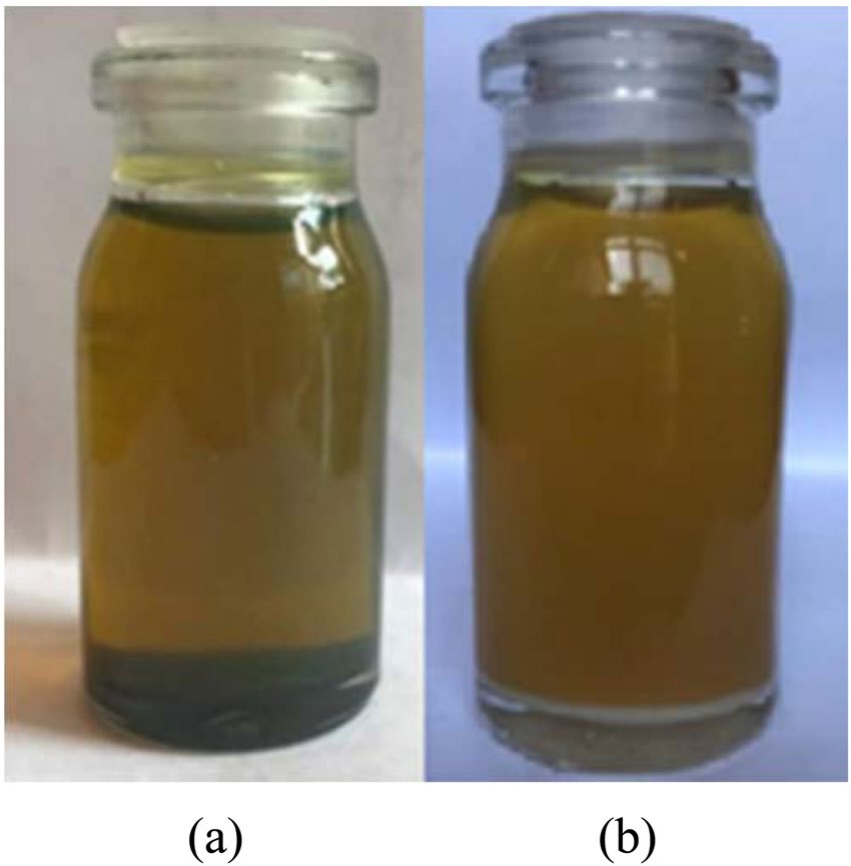
Camera pictures of nano lubricants (at 0.7 wt.%) (**a**) MoS_2_ after 1 day, (**b**) ZnO after 3 days.

Figure 9(a) shows the stability of MoS_2_ nano lubricant at 0.7% that was taken after 1 day at room temperature. After 12 hours, sedimentation started slowly for 0.7 wt.% of MoS_2_ nano lubricant, and gradually nanoparticles tend to settle. Figure 9(b) presents the stability of ZnO nano lubricant at 0.7 wt.% that is taken at room temperature after 3 day. The ZnO nano lubricant at 0.7 wt.% was stable for at least 3 days so that no precipitation occurs during this time. The density of the prepared samples was also measured for two weeks, and no significant change was observed in the densities of 0.1 wt.% and 0.4 wt.% of both nano lubricants after 2 weeks.

### Viscosity

**Figure 10:**
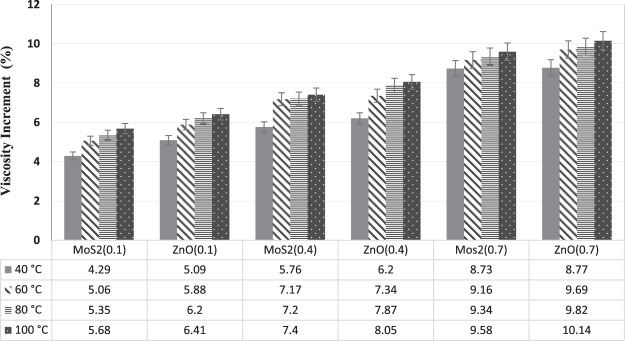
Viscosity increment of ZnO and MoS_2_ nano lubricants at diverse concentrations and temperatures.

Viscosity increment of prepared ZnO and MoS_2_ nano lubricants in different concentrations (0.1, 0.4 and 0.7 wt.%) of nano-additives and various temperatures (40, 60, 80 and 100 °C) are presented in Fig. 10.

The mathematical relation of viscosity measurement is represented in Eq. (17)

17 $${\rm{Viscosity}}\,{\rm{increment}}( \% )=\frac{{\rm{nanofluid}}\,{\rm{viscosity}}-{\rm{pure}}\,{\rm{diesel}}\,{\rm{oil}}\,{\rm{viscosity}}}{{\rm{pure}}\,{\rm{diesel}}\,{\rm{oil}}\,{\rm{viscosity}}}\times 100$$

Concerning Fig. 10, it can be seen that the kinematic viscosity of all samples that contain nanoparticles, has increased in comparison with the base fluid even in the lower concentrations, and in higher concentrations, the increase was more tangible. As the temperature rises, the viscosity of all samples was decreased. The highest viscosity increase was observed at 0.7 wt.% and 100 °C for each of the ZnO and MoS_2_ nanoparticles, which is 10.14 and 9.58%, respectively. The placement of nanoparticles between the oil layer leads to an increase of viscosity. The agglomeration of nanoparticles and the formation of larger and asymmetric particles in higher concentrations also increase collisions which increase the nano lubricants viscosity relative to the base fluid. The reduction of molecular forces between the base fluid and the surface of the nanoparticles due to the increase in the speed of the nanoparticles decreases the viscosity at high temperatures. At all measured temperatures, ZnO nanoparticles had higher viscosity values than MoS_2_ nano lubricant because of the higher accumulation of ZnO nanoparticles and the close proximity of nanoparticles to MoS_2_, which makes the ZnO nanoparticles more coherent. Also, the ZnO nano lubricant, due to its better stability than MoS_2_ nano lubricant, leads to a higher viscosity in the same percentages of weight per nano lubricant.

**Figure 11:**
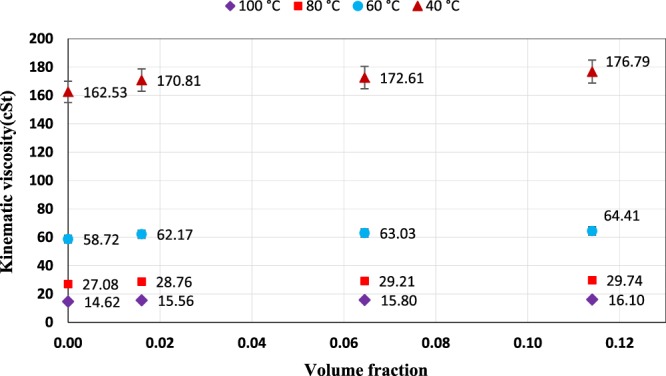
Kinematic viscosity as a function of volume fraction for ZnO nano lubricants.

**Figure 12:**
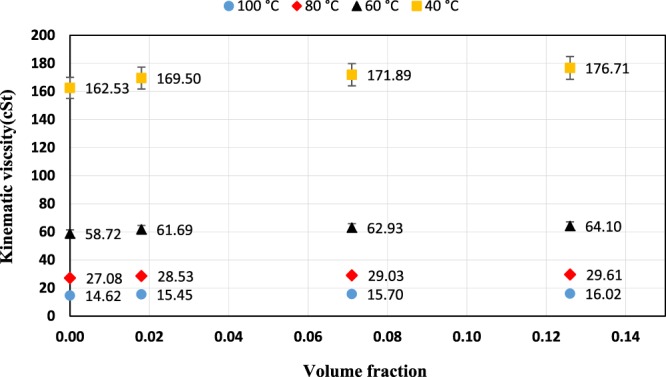
Kinematic viscosity as a function of volume fraction for MoS_2_ nano lubricants.

Variation of kinematic viscosity of ZnO and MoS_2_ nanoparticles as a function of volume fraction at various temperatures are shown in Figs. 11 and 12, respectively. As seen in the figures, the nano lubricant viscosity increased with an increase in volume fraction at a constant temperature. The reason for the increase of viscosity can be described as one of the factors that affect the nano lubricant viscosity, the random motion of nanoparticles within the base fluid, and the continuous collisions of these particles with the base fluid molecules. In addition, when nanoparticles are added to the base fluid, the Van der Waals force between the nanoparticles and the base fluid causes agglomeration of the nanoparticles that these agglomerates prevent the movement of base fluid’s molecules, which results in increased viscosity. The volume fractions were calculated by the following equation:

18 $${\rm{\varphi }}=\frac{{{\rm{m}}}_{{\rm{p}}}/{{\rm{\rho }}}_{{\rm{p}}}}{{{\rm{m}}}_{{\rm{p}}}/{{\rm{\rho }}}_{{\rm{p}}}+{{\rm{m}}}_{{\rm{f}}}/{{\rm{\rho }}}_{{\rm{f}}}}\times 100$$

Here, φ is the volume fraction of nanoparticles, m_p_ and m_f_ are the mass of nanoparticles and the base fluid, ρ_p_, and ρ_f_, are the density of the nanoparticles and base fluid. In Table 2, the obtained values of the volume fraction are presented.

**Table 2:** Volume fractions equal to the used weight fraction.

Weight fraction	0.1	0.4	0.7
Volume fraction (ZnO)	0.016	0.0645	0.114
Volume fraction (MoS_2_)	0.018	0.071	0.126

### The relative viscosity of nano lubricants

**Figure 13:**
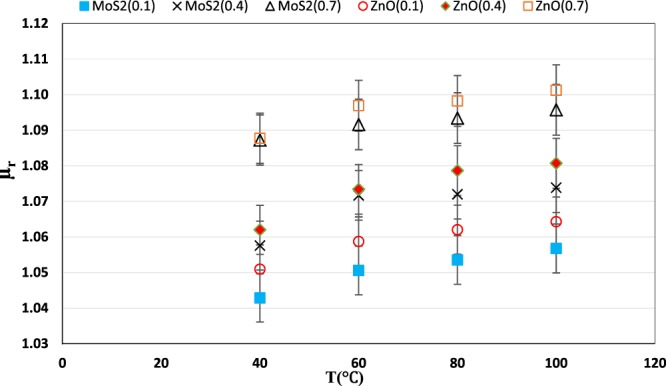
Variation of relative viscosity at different temperatures.

Figure 13 shows the relative viscosity $({\mu }_{{\rm{r}}}=\frac{{\mu }_{{\rm{nf}}}}{{\mu }_{{\rm{bf}}}})$ changes with temperature in different mass fractions for ZnO and MoS_2_ nanoparticles. The figure shows that in each temperature, the relative viscosity of the ZnO nano lubricant is higher than the MoS_2_ nano lubricant. The maximum increase of nano lubricant’s relative viscosity compared to the base fluid at 100 °C and 0.7 wt.% for both ZnO and MoS_2_ nano lubricant was 1.101 and 1.095, respectively.

### Viscosity index

Viscosity index (VI) is another important parameter in determining the properties of lubricants, which is obtained from kinematic viscosity at temperatures of 40 °C and 100 °C. Table 3 shows the changes in the viscosity index by adding nanoparticles. According to the table, the viscosity index increased with the addition of nanoparticles. At the same concentrations of each nanoparticle, the viscosity index of ZnO nano lubricant was more than MoS_2_ nano lubricant. The maximum increase was observed for ZnO and MoS_2_ nano lubricant at a concentration of 0.7 wt.%, which was 7.88% and 7.04%, respectively. By increasing the kinematic viscosity of nano lubricant at two temperatures of 40 °C and 100 °C, the viscosity index was also increased. With increasing ZnO nano lubricant kinematic viscosity relative to the MoS_2_ nano lubricant, the increase in the viscosity index was also higher in the presence of ZnO nanoparticles and it indicated that with temperature changes, the thermal behavior of the ZnO nano lubricant was more predictable than MoS_2_ nano lubricant.

**Table 3:** Variations of viscosity index for different concentrations of ZnO and MoS_2_ nano lubricants.

Concentration (wt.%)	Viscosity Index	Viscosity Index enhancement (%)
0 (pure oil)	86.85	—
0.1 (ZnO)	91.87	5.79
0.4 (ZnO)	93.24	7.36
0.7 (ZnO)	93.69	7.88
0.1 (MoS_2_)	91.53	5.39
0.4 (MoS_2_)	92.68	6.71
0.7 (MoS_2_)	92.96	7.04

### Flash point and pour point

**Figure 14:**
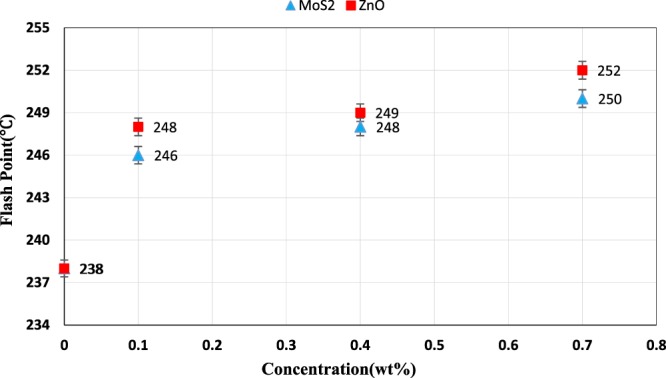
Flash point of ZnO and MoS_2_ nano lubricants at different concentrations.

Pour point is the lowest temperature at which the oil can flow in that state and the flash point is the lowest temperature at which the oil is sufficiently converted to steam and it creates a flammable mixture with air, that represents the maximum and minimum operating temperature of the oil. Figure 14 shows the effect of nanoparticles concentration on the flash point. According to the figure, for both nano lubricants, the flash point increased with increasing the nanoparticles concentration, which indicates an increase in the upper limit of the operating temperature of the oil. The maximum increase for each nano lubricant was at 0.7 wt.%. For nano lubricants containing 0.7 wt.% ZnO and MoS_2_, 5.04 and 5.88% increase in flash point were seen, respectively. Thus, the operating temperature of the ZnO nano lubricant was higher than the MoS_2_ nano lubricants. Which is due to the better thermophysical properties and better stability of ZnO nanoparticles than those of MoS_2_ nanoparticles. Generally, the flammable resistance of the nano lubricants can be ascribed to an augment in the thermal conductivity of the nano lubricants due to the addition of the nanoparticles. Hence, the enhanced flash point can be considered as an advantage in relation to enhanced lubricity characteristics of pure oil.

**Figure 15:**
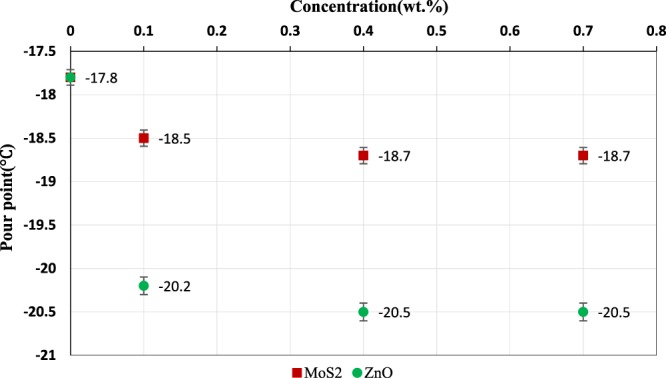
Variation of pour point at diverse concentrations of the MoS_2_ and ZnO nano lubricants.

The effect of adding nanoparticles at different concentrations on the pour point is shown in Fig. 15. Considering Fig. 15, it is noted that the addition of nanoparticles decreased the pour point. The reduction of the pour point in the presence of ZnO nanoparticles was more than that of MoS_2_ nanoparticles, which is due to better thermophysical properties of ZnO nanoparticles rather than MoS_2_ nanoparticles. The optimum concentration for both nanoparticles was 0.4 wt.%. Temperature reduction decreases proper movement of nanoparticles; furthermore, the effectiveness of the nanoparticles decreases due to the agglomeration of the nanoparticles at higher concentrations.

### Tribological tests results on pin-on-disc tribometer

**Figure 16:**
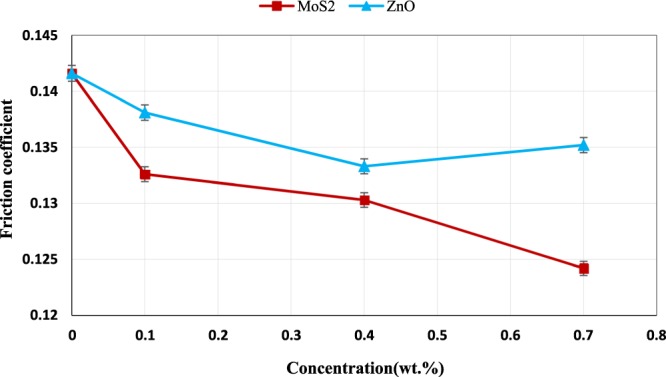
Variation of the friction coefficient as a function of nanoparticles concentration.

The average friction coefficient values as a function of nanoparticles concentration are presented in Fig. 16. Considering the Fig. 16, it is noted that the addition of ZnO and MoS_2_ nanoparticles decreased friction coefficient values. By comparing the recorded friction coefficient values for both nano lubricants at identical concentrations, it can be concluded that the MoS_2_ nanoparticles had a better friction-reduction function relative to the ZnO nanoparticles. It is due to the structural properties of the MoS_2_ nanoparticles with extensive space and weakly bonded Van der Waals forces between the S-Mo-S sandwich layers and the pure positive charge on the surface that leads to the spread of electrostatic repulsion. So, the layers are placed together with weak molecular forces and can easily slip over each other. It was also observed that friction coefficient values of MoS_2_ nano lubricant decreased with increasing nanoparticle concentrations, however, the friction coefficient of ZnO nano lubricants decreased first and then increased with increasing content of ZnO nanoparticles. This can be attributed to the fact that ZnO nanoparticles were agglomerated at high concentrations that prevent their effective and correct functioning. Therefore, the optimal concentration for MoS_2_ and ZnO nano lubricants was 0.7 wt.% and 0.4 wt.%, respectively. Compared to the base fluid, the friction coefficient was reduced by 12.29% and 5.86% for MoS_2_ and ZnO nano lubricants at the optimal concentration of each, respectively. Regarding Fig. 16, it is noted that there was no significant reduction in friction coefficient with the addition of nanoparticles, which can be due to the high viscosity of the base fluid, which despite the high force applied at a distance of 1000 m, prevented the effective performance of the nanoparticles in reducing the friction coefficient. In order to maintain good stability and better anti-friction feature, nanoparticles need to be modified with a proper surfactant, so it may be possible that the used surfactant was not effective. The tribological characteristic of nanoparticles in any base oil needs to be completely evaluated and scrutinized. Furthermore, the entrance of the nano additives in the diesel oil near the friction pairs into the contact area is an important phenomenon. If the nano additives in the diesel oil cannot enter into the contact area, it would prevent the proper anti-wear function of the system. Moreover, the form and the amount of particles entering the contact area are other crucially significant identification factors in this phenomenon. Actually, the issue of how the form and size of nanoparticles benefit in achieving lubrication behavior is still not known. As research to explain such mechanisms provides the proper utilization of nano additives in gears, rolling bearings, and engines, it should be very attractive and important if the fundamental mechanism involved in the mode of nanoparticles entering the contact area is properly revealed^67–71^.

The most significant factors that can improve the lubrication characteristics of the nano lubricants are size, shape, morphology, and the intrinsic properties of the nano additives. The lower the diameter of the nano additives, the better the tribological properties. However, in this study, the most important parameter in improving the anti-wear and anti-friction behavior of the nano lubricants is the structure of the MoS_2_ nanoparticles. Because it shears simply under sliding contact owing to consisting of vertically stacked, weakly interacting layers held together by Van der Waals forces, giving rise to a low friction coefficient, MoS_2_ are widely applied as additives in the oil. It is worth mentioning that, the MoS_2_ nano lubricants are less likely to form an agglomeration compared to the ZnO nanoparticles. On the other hand, in improving the thermophysical feature, ZnO nanoparticles are better in comparison with MoS_2_ nanoparticles. Although ZnO nanoparticles have a greater effect on these properties owing to their inherent characteristics, ZnO nanoparticles are prone to be agglomerated due to their high surface energy which can be the other important factor.

**Figure 17:**
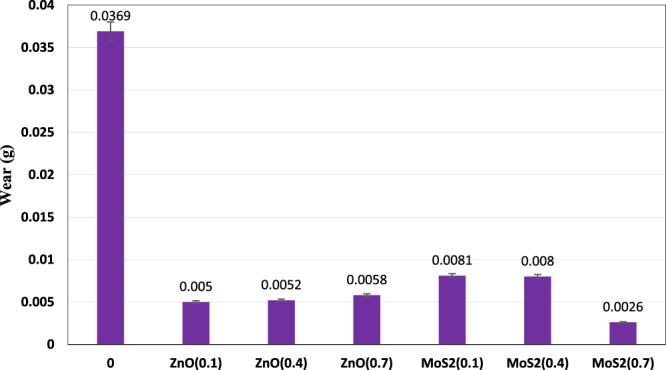
Mass loss (g) of the pins at diverse concentrations.

Figure 17 shows the average mass loss of pins after the pin-on-disc test for all samples. Considering the Fig. 17, it was observed that the addition of nanoparticles even at low concentrations leads to a reduction in the wear of the samples. Wear were reduced by 86.48, 85.91 and 84.28% for 0.1, 0.4 and 0.7 wt.% ZnO nano lubricants, respectively and by comparison with the reduced wear for MoS_2_ nano lubricants at concentrations of 0.1, 0.4 and 0.7 wt.%, which were 78.05%, 78.32% 92.95%, it can be concluded that the MoS_2_ nano lubricants had a better performance than the ZnO nano lubricants at higher concentrations. For ZnO nano lubricants, wear increased with increasing concentration, which can be due to the agglomeration of ZnO nanoparticles at high concentrations, which prevented the proper functioning of the system.

### Worn surfaces

**Figure 18:**
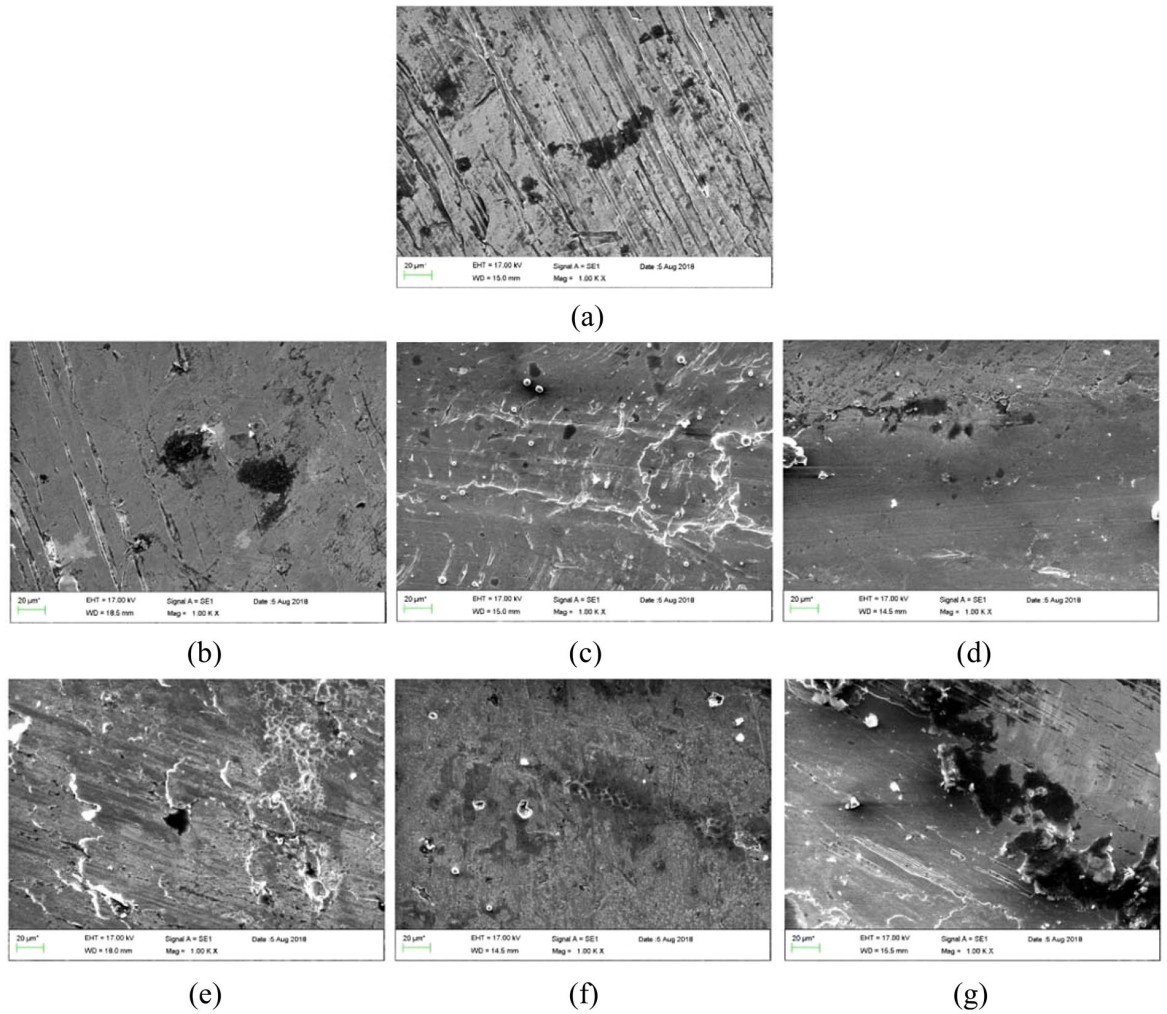
SEM morphologies of the worn surfaces lubricated with (**a**) pure diesel oil, (**b**) 0.1 wt.% MoS_2_ nano lubricants, (**c**) 0.4 wt.% MoS_2_ nano lubricants, (**d**) 0.7 wt.% MoS_2_ nano lubricants, (**e**) 0.1 wt.% ZnO nano lubricants, (**f**) 0.4 wt.% ZnO nano lubricants, (**g**) 0.7 wt.% ZnO nano lubricants.

In order to figure out the lubricity characteristic, the worn surfaces were investigated by SEM. Figure 18 exhibits the images of the worn surfaces lubricated by pure diesel oil and nano lubricants at different concentrations under load (75 N) and speed (150 rpm) for 1000 m. According to Fig. 18, it was observed that the images related to nano lubricant have fewer scratches and a smoother surface than the base fluid, in other words, anti-wear and anti-friction properties have improved in the presence of ZnO and MoS_2_ nanoparticles. The nano lubricant prevents direct contact of the rubbing surface by forming a protective layer. Considering the images of the MoS_2_ nano lubricant, it is noted that with increasing concentration, the scratches are less, However, for ZnO nano lubricant, these scratches have been reduced first, but then have been increased at a concentration of 0.7 wt.%, which can be due to agglomeration of ZnO nano lubricant at high concentrations. Also, by comparing the images of MoS_2_ and ZnO nano lubricants, it can be concluded that the MoS_2_ nano lubricant had better tribological properties than the ZnO nano lubricant.

### Lubrication mechanism

**Figure 19:**
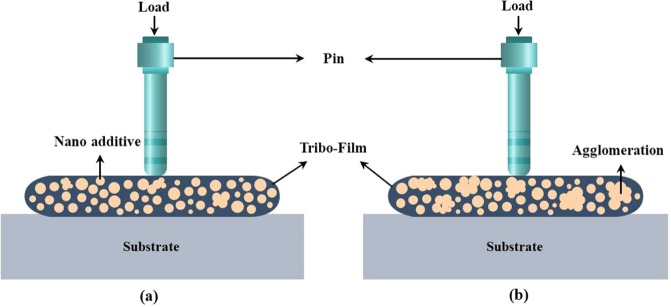
Schematic illustration of lubrication mechanism of (**a**) nano lubricants tribo-film with low agglomeration and proper functioning, (**b**) nano lubricants tribo-film at higher concentration including greater agglomeration.

Figure 19 schematically shows the lubrication mechanism of the prepared nano lubricants at room temperature. Figure 19(a) illustrates that when MoS_2_ and ZnO nanoparticles are added into base oil used as lubricant nano additive; the main lubrication mechanism is that the formation of the transfer film due to the relative sliding of the nanoparticles at both counterparts. The tribo-film created and the practical MoS_2_ and ZnO nanoparticles supplied into the contact area are profoundly affected in the development of tribological characteristics. With increasing the concentration of ZnO nano lubricant from 0.4 wt.%, some ZnO nanoparticles are agglomerated, and thus, the secondary particle size becomes larger as shown in Fig. 19(b). This would worsen the friction and wear, and consequently lead to an increment in COF and wear area. This means that the 0.4 wt.% can be acknowledged as the optimal concentration of ZnO nano lubricants. The 0.4 wt.% ZnO nano lubricant can provide sufficient nanoparticles continuously to the contact area with little agglomeration. Hence, 0.4 wt.% is considered to be the optimal mass fraction of ZnO as the most excellent tribological attributes including the lowest COF and the most effective wear resistance have been received. For ZnO nano lubricant below 0.1 wt.%, it provides only limited ZnO nanoparticles to take effect, while the lubricant with a high ZnO concentration of 0.4 wt.% indicates the ZnO nanoparticles to agglomerate. On the other hand, the agglomeration of ZnO nanoparticles operates as a barrier to hinder the perpetual supplies of fine nanoparticles to the contact zone for lubrication. In contrast, the agglomerated nanoparticles would abrade the pin and disk surfaces to a great extent. Hence, the lubricant containing 0.4 wt.% ZnO manifests the most desirable lubricating behavior. For MoS_2_ nano lubricants, as can be seen in Fig. 16, the COF decrease with the addition of MoS_2_ nanoparticles. It indicates that MoS2 nanoparticles are not prone to be agglomerated during the test; moreover, they can supply a proper amount of nano lubricants continuously to the contact area. In nutshell, MoS_2_ nanoparticles had more excellent anti-friction and anti-wear characteristics in comparison with ZnO nanoparticles^4,29,53,72,73^.

### Friction factor

**Figure 20:**
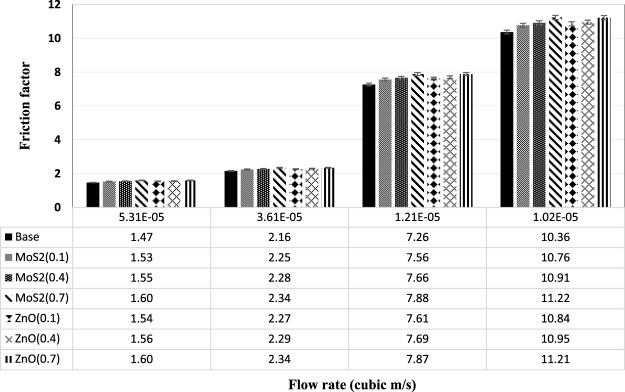
Variation of friction factor as a function of flow rate at diverse concentrations.

**Figure 21:**
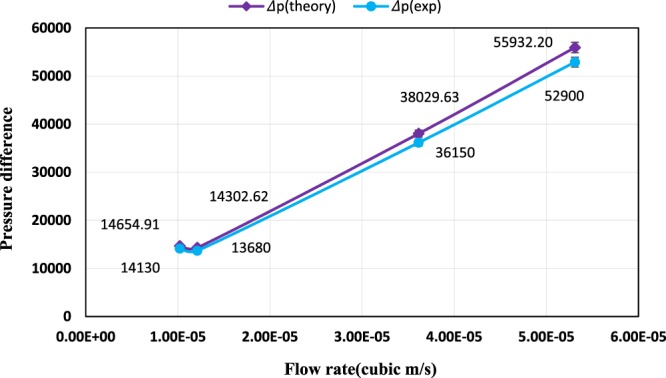
Variation of pressure difference as a function of flow rate for the pure diesel oil.

By using a designed laboratory system, experiments were carried out at diverse flow rates and Reynolds numbers and friction factor values were calculated at these flow rates. The friction factor variations in terms of flow rate for ZnO and MoS_2_ nanoparticles are presented in Fig. 20. Regarding the figure, it can be seen that the friction factor decreased with increasing flow rate. In similar flow rates of each passing fluid from the tube, higher values of friction factor were observed compared to the pure diesel oil due to the presence of nanoparticles. Considering that in the same concentrations, the kinematic viscosity of ZnO nano lubricant was higher than MoS_2_ nano lubricant, so the friction factor of ZnO nano lubricant was higher than the MoS_2_ nano lubricant. Stability also has a direct impact on the friction factor, so that the agglomeration of nanoparticles, particularly in high concentrations, prevents the proper functioning of the systems in contact. Figure 21 shows the experimental and theoretical pressure difference values at various flow rates for pure diesel oil. According to the low discrepancy between experimental and theoretical pressure, the correct performance of setup can be achieved.

## Conclusions

In the present study, the comparison of the thermophysical and tribological properties of nano-ZnO and nano-MoS_2_ as additives in diesel oil was investigated. For this purpose, with the synthesis of ZnO and MoS_2_ nano lubricants, the thermophysical and tribological properties of the prepared nano lubricant in various formulations have been investigated. The results of the research are as follows:

1. In the study of the kinematic viscosity of prepared nano lubricants at different temperatures and concentrations and compared with the base fluid, it was observed that the addition of nanoparticles to base fluid increases the viscosity. Also, the increase in viscosity increased with increasing nanoparticles concentration, and the effect of increasing the ZnO nanoparticles was higher than that of MoS_2_ nanoparticles.
2. By adding ZnO and MoS_2_ nanoparticles to the pure fluid, the viscosity index increased and in higher concentrations, this increase was higher.
3. Adding nanoparticles to the pure fluid decreased the pour point. The optimum concentration for both nano lubricants is also 0.4 wt.%. Also, by adding more than 0.4 wt.% of the nanoparticles, no changes in the pour point have been observed.
4. Adding ZnO and MoS_2_ nanoparticles to pure oils at different concentrations increased the flash point of the nano lubricants relative to the pure diesel oil. Compared to the pure diesel oil, the flash point of ZnO and MoS_2_ nano lubricants at 0.7 wt.% of each of the nanoparticles increased by 5.88% and 5.04%, respectively.
5. The pure diesel oil containing ZnO and MoS_2_ nanoparticles showed better anti-friction and anti-wear features as compared with the pure lubricant. The optimum concentration for ZnO and MoS_2_ nanoparticles was 0.4 wt.% and 0.7 wt.%, respectively. Friction coefficient at optimal concentrations from each of the ZnO and MoS_2_ nano lubricants decreased by 5.86% and 12.29%, respectively. Also, the results of the mass loss of the pins indicated better anti-wear performance of MoS_2_ nanoparticles than ZnO nanoparticles.
6. By investigating the effect of nanoparticle concentrations on the friction factor at different flow rates, it was observed that the friction factor increased with increasing nanoparticle concentrations at the same flow rate.
